# Causes and predictors of unplanned reoperations within 30 days post laparoscopic pancreaticoduodenectomy: a comprehensive analysis

**DOI:** 10.3389/fonc.2024.1464450

**Published:** 2024-08-23

**Authors:** Shiwei Zhang, Dipesh Kumar Yadav, Gaoqing Wang, Yin Jiang, Jie Zhang, Rajesh Kumar Yadav, Alina Singh, Guo Gao, Junyu Chen, Yefan Mao, Chengwei Wang, Yudi Meng, Yongfei Hua

**Affiliations:** ^1^ Department of Hepatobiliary and Pancreatic Surgery, Ningbo Medical Center Lihuili Hospital, Ningbo University, Ningbo, Zhejiang, China; ^2^ Department of General Surgery, Wenzhou People’s Hospital, The Third Clinical Institute Affiliated to Wenzhou Medical University, The Third Affiliated Hospital of Shanghai University, Wenzhou, China; ^3^ College of Pharmacy, University of Louisiana at Monroe, Monroe, LA, United States; ^4^ Department of Surgery, Parkland Medicare and Research Center, Janakpur, Nepal

**Keywords:** pancreatic tumor, laparoscopic pancreaticoduodenectomy, unplanned reoperation, risk factors, postoperative hemorrhage

## Abstract

**Objective:**

To delineate the risk factors and causes of unplanned reoperations within 30 days following laparoscopic pancreaticoduodenectomy (LPD).

**Methods:**

A retrospective study reviewed 311 LPD patients at Ningbo Medical Center Li Huili Hospital from 2017 to 2024. Demographic and clinical parameters were analyzed using univariate and multivariate analyses, with P < 0.05 indicating statistical significance.

**Results:**

Out of 311 patients, 23 (7.4%) required unplanned reoperations within 30 days post-LPD, primarily due to postoperative bleeding (82.6%). Other causes included anastomotic leakage, abdominal infection, and afferent loop obstruction. The reoperation intervals varied, with the majority occurring within 0 to 14 days post-surgery. Univariate analysis identified significant risk factors: diabetes, liver cirrhosis, elevated CRP on POD-3 and POD-7, pre-operative serum prealbumin < 0.15 g/L, prolonged operation time, intraoperative bleeding > 120 ml, vascular reconstruction, soft pancreatic texture, and a main pancreatic duct diameter ≤3 mm (all P < 0.05). Multivariate analysis confirmed independent risk factors: pre-operative serum prealbumin < 0.15 g/L (OR = 3.519, 95% CI 1.167-10.613), CRP on POD-7 (OR = 1.013, 95% CI 1.001-1.026), vascular reconstruction (OR = 9.897, 95% CI 2.405-40.733), soft pancreatic texture (OR = 5.243, 95% CI 1.628-16.885), and a main pancreatic duct diameter ≤3 mm (OR = 3.462, 95% CI 1.049-11.423), all associated with unplanned reoperation within 30 days post-LPD (all P < 0.05).

**Conclusion:**

Postoperative bleeding is the primary cause of unplanned reoperations after LPD. Independent risk factors, confirmed by multivariate analysis, include low pre-operative serum prealbumin, elevated CRP on POD-7, vascular reconstruction, soft pancreatic texture, and a main pancreatic duct diameter of ≤3 mm. Comprehensive peri-operative management focusing on these risk factors can reduce the likelihood of unplanned reoperations and improve patient outcomes.

## 1 Introduction

Gagner and Pomp pioneered the technique of laparoscopic pancreaticoduodenectomy (LPD) in 1994 (1). Subsequently, numerous medical institutions globally have incorporated LPD into their standard procedures for addressing both benign and malignant pancreatic head tumors, periampullary cancers and distal cholangiocarcinoma (2). Despite the procedure’s reputation for being technically demanding due to the constraints of laparoscopic instruments in confined spaces, the absence of tactile feedback, the challenge of managing hemorrhage during major vascular injuries, the complexity of biliary and pancreatic reconstruction, and the necessity of adhering to oncological principles during tumor resection, the safety and feasibility of LPD have been well-documented in prior research. Studies have highlighted that LPD offers several advantages over open surgery, including smaller incisions, reduced blood loss, decreased postoperative discomfort, shorter hospital stays, a lower rate of delayed gastric emptying (DGE), and expedited recovery (3, 4). Nevertheless, all these specific aspects typically require a surgeon with a high level of surgical expertise, and who is well-versed in minimally invasive surgical techniques.

Advances in laparoscopic technology have led to a significant reduction in the peri-operative mortality rate associated with LPD, ranging from 0% to 4.8% (5, 6). Nonetheless, the rate of post-operative complications remains substantial, between 29% and 68.8% (5–8). Complications such as pancreatic fistula, intra-abdominal abscess, bile leak, and postoperative hemorrhage can lead to severe outcomes and necessitate unplanned readmission and additional surgery.

In recent times, unplanned readmission and reoperation have been recognized as critical indicators of the quality of post-operative care and surgical outcomes, significantly impacting patient prognosis and healthcare service quality. Lately, multiple organizations including The Joint Commission of the USA have emphasized the importance of using unplanned reoperation as a measure of surgical quality and have encouraged thorough documentation and reporting of such incidents (9, 10). Although it is widely acknowledged that reoperation is a significant factor contributing to secondary injury, extended hospital stays, increased financial burden, and heightened medical workload, there is a dearth of literature on the risk factors and prevalence of unplanned reoperation following LPD.

This study aims to meticulously identify the risk factors, causes, and incidence of unplanned reoperation within 30 days post-LPD, providing a comprehensive understanding of peri-operative management with the goal of enhancing surgical safety, improving patient outcomes, and mitigating surgical risks.

## 2 Materials and methods

### 2.1 Patients and data

We conducted a retrospective review of clinical data for all patients who underwent LPD at Li Huili Hospital, affiliated to Ningbo University, from February 2017 to May 2024. Eligibility for inclusion was based on two criteria (1): patients who underwent LPD, and (2) those with comprehensive clinical records. The exclusionary factors were as follows (1): patients who initially had an open pancreaticoduodenectomy (2), those who were initially intended for LPD but later required conversion to an open procedure (3), patients with concurrent conditions necessitating surgical intervention, such as acute gastrointestinal hemorrhage induced by pancreatic head cancer or bleeding from a duodenal tumor, and (4) individuals with incomplete clinical data.

The study received approval from the Medical Ethics Committee of Li Huili Hospital, affiliated to Ningbo University, under the reference number KY2024Sl215, and adhered to the principles outlined in the Declaration of Helsinki (11). Additionally, informed consent was obtained from all participants or their legal representatives prior to surgery, authorizing the collection and utilization of their medical data for research purposes.

Our study encompassed a total of 311 patients (169 males and 142 females) who underwent LPD. Patients data were systematically extracted from Electronic Medical Records (EMRs) and organized into a standardized Excel format. The collected variables included demographic information, such as gender, age, body mass index (BMI), disease-related data including pre-operative nutritional status, pre-operative comorbidities, other disease history, pre-operative American Society of Anesthesiologists (ASA) risk grading (12), and pre-operative laboratory values, history of prior upper abdominal surgeries, and tumor characteristics such as tumor type, location, size, nerve invasion, and lymph node metastasis. Additionally, we documented surgical details including the surgical approach, blood loss during the procedure, peri-operative blood transfusion volume, number of lymph nodes resected, and the diameter of the main pancreatic duct, and postoperative outcomes such as the use of somatostatin, occurrence of complications, and the need for unplanned reoperations.

### 2.2 Per-operative evaluation

**Figure 1:**
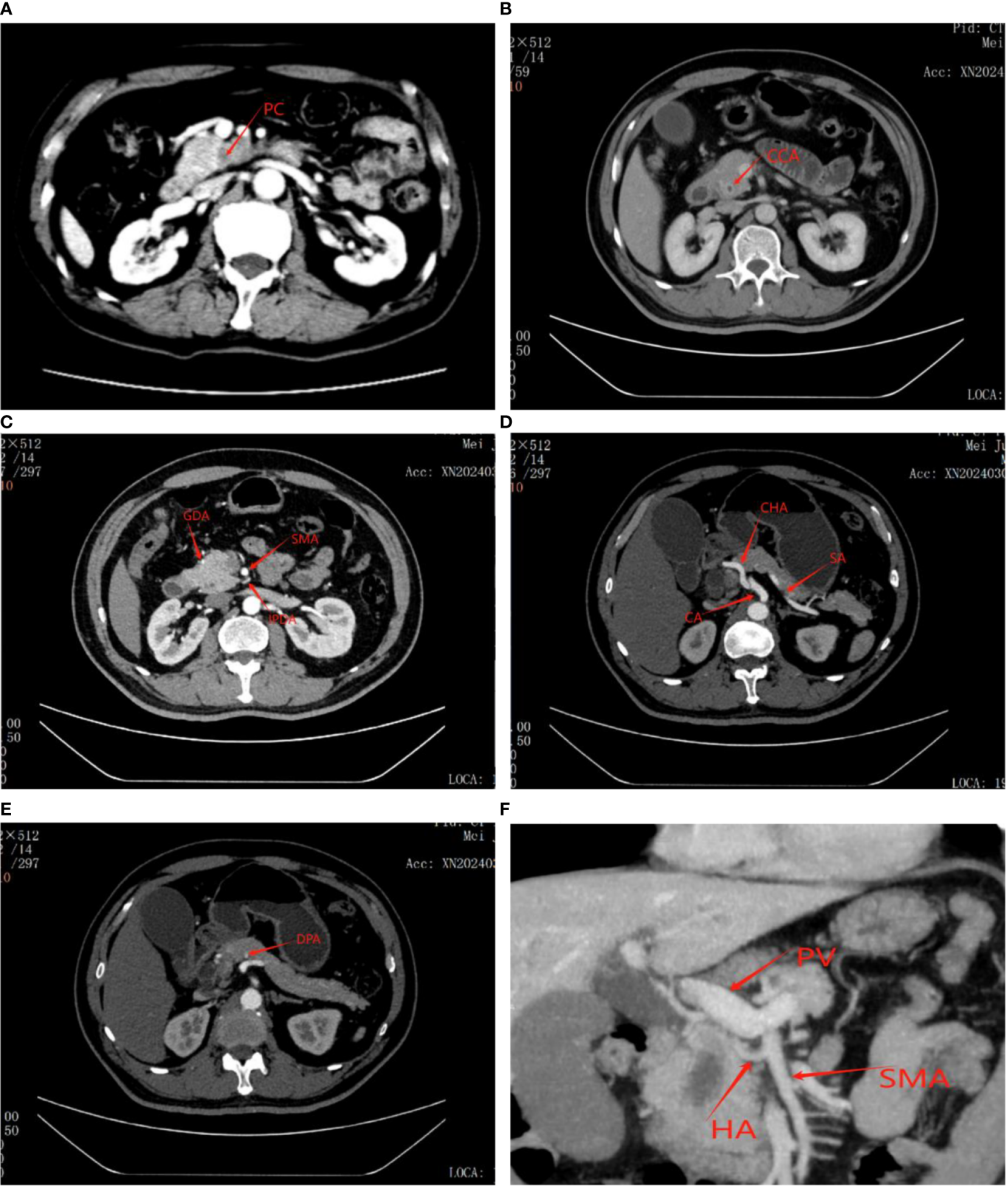
Figure showing tumor as well as its relationship to surrounding blood vessels and organs on CT scan before laparoscopic pancreaticoduodenectomy. **(A)** Pancreatic cancer. **(B)** Distal bile duct cancer. **(C)** GDA, Gastroduodenal artery; SMA, Superior mesenteric artery; IPDA, Inferior pancreaticoduodenal artery. **(D)** CA, Celiac artery; CHA, Common hepatic artery; SA, Splenic artery. **(E)** DPA, Distal pancreatic artery. **(F)** Accessory or replaced right hepatic artery (HA); PV, Portal Vein.

Prior to surgery, all patients underwent a comprehensive pre-operative evaluation involving biochemical tests and imaging studies to determine their suitability for the procedure. Specifically, contrast-enhanced abdominal computed tomography (CECT) and magnetic resonance imaging (MRI) were utilized to meticulously examine the lesion, assessing its characteristics, including nature, location, and size, as well as its relationship to surrounding blood vessels and organs (**Figure 1**). Furthermore, in the case of complex or challenging situations, they were thoroughly discussed during our multidisciplinary team meetings, ensuring a well-informed and collaborative approach to pre-surgical planning.

### 2.3 Surgical techniques

**Figure 2:**
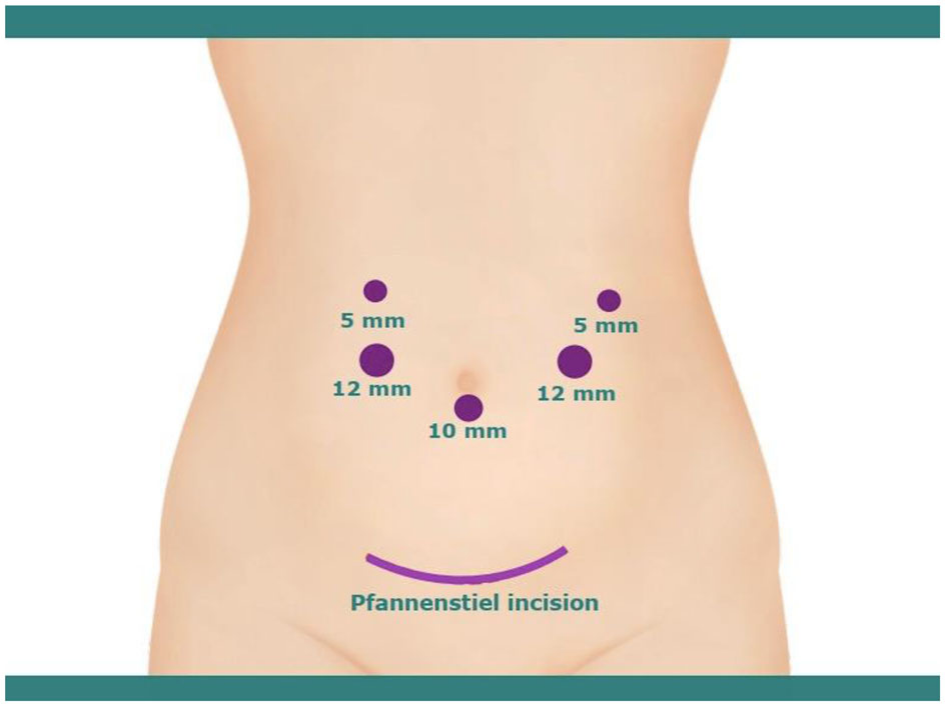
Illustration of trocar positioning for laparoscopic pancreaticoduodenectomy and pfannenstiel incision for removal of specimen.

The LPD was performed under general anesthesia. To begin with, the patient was positioned supine in the anti-Trendelenburg position, with the patient’s arms resting alongside the body to ensure ample working space for the surgical team, which includes the surgeon, assistant, and scrub nurses. Furthermore, the patient’s legs were spread to accommodate the camera operator, who was positioned in the space between the legs. The surgical procedure was conducted using the ‘five-hole method,’ with the operating surgeon stationed on the right side of the patient (**Figure 2**).

#### 2.3.1 Dissection

In brief, a thorough laparoscopic exploration of the abdominal cavity was conducted to rule out any distant metastasis. Upon confirmation of the absence of metastasis, the gastrocolic ligament was meticulously dissected below the gastroepiploic vessels using a harmonic scalpel to access the lesser sac, with careful attention paid to avoid damage to the transverse mesocolic vessels. The infra-pancreatic region was then carefully dissected to expose the superior mesenteric vein (SMV) and portal vein (PV) up to the superior border of the pancreas, creating a posterior pancreatic tunnel just below the neck of the pancreas. During this stage of dissection, the gastroepiploic vein was ligated if it was draining separately into SMV, otherwise it was preserved in a case where gastroepiploic vein and superior right colic vein combined to form the gastrocolic trunk of Henle.

**Figure 3:**
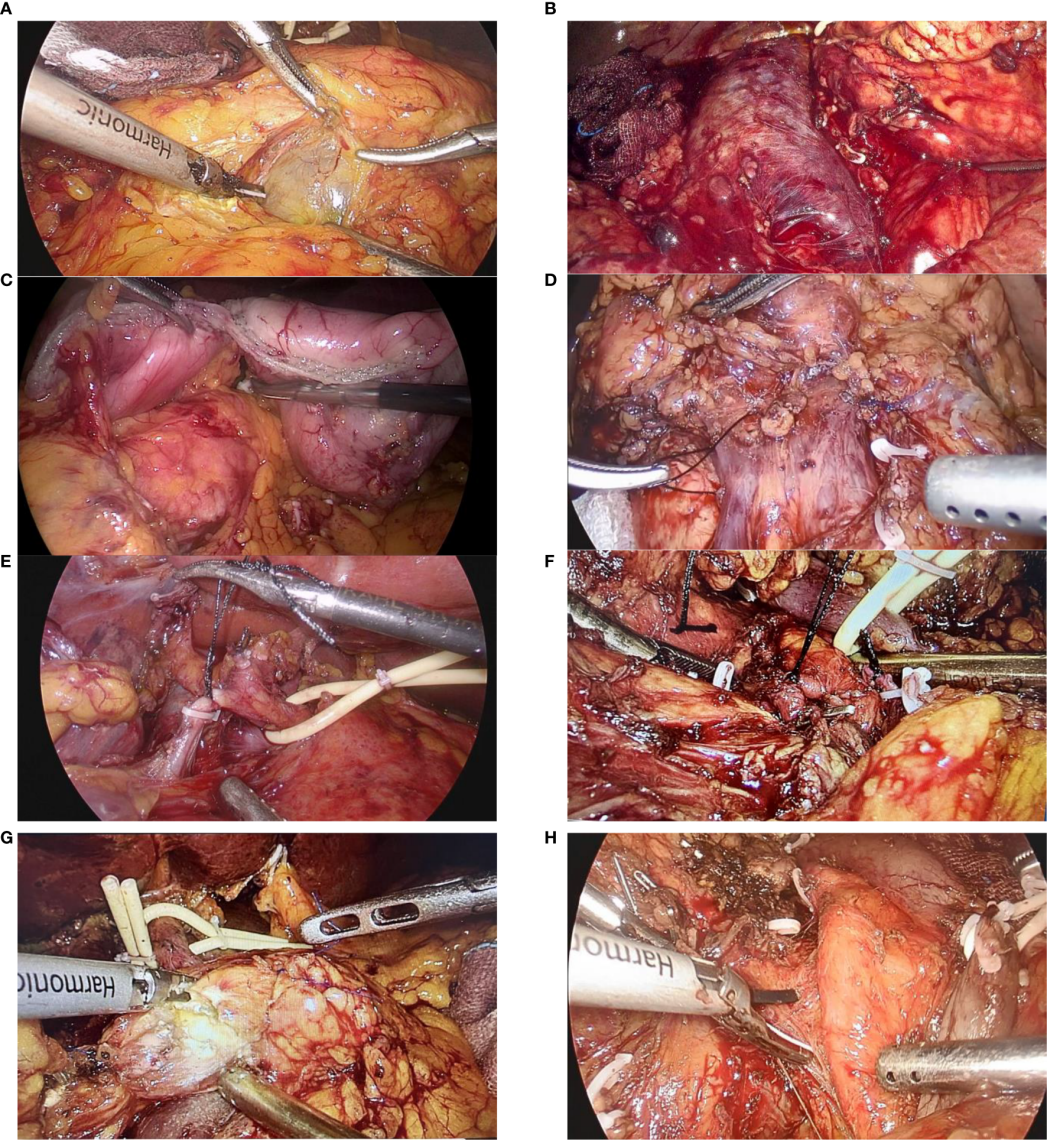
Figure showing the various steps of dissection during a laparoscopic pancreaticoduodenectomy: **(A)** Exposure of the infra-pancreatic superior mesenteric vein; **(B)** Kocher’s maneuver, with exposure of the inferior vena cava (IVC) and left renal vein; **(C)** Transection of the duodenum and stomach; **(D)** Ligation of the gastrocolic trunk of Henle; **(E)** Ligation of the gastroduodenal artery (GDA); **(F)** Ligation of the inferior pancreaticoduodenal artery (IPDA); **(G)** Transection of the pancreatic head from the neck; **(H)** Separation of the superior mesenteric vein (SMV) and superior mesenteric artery (SMA) from the pancreatic head and uncinate process.

Besides, the dissection was carried at the hilar area of the liver, where the hepatoduodenal ligament was then dissected judiciously in order to free the proper hepatic artery, common bile duct, and PV. The dissection was further advanced to expose the common hepatic artery and the gastroduodenal artery (GDA), where GDA was then ligated and divided. Lymph nodes in the vicinity of the common hepatic artery were harvested for a frozen section analysis. Additionally, the gallbladder was resected and the common hepatic duct was then transected using scissors. To avoid any spillage of bile, the stump of the common hepatic duct was clamped with the bulldog clamp. Furthermore, the hepatogastric ligament was dissected to free the distal stomach and the pylorus, which were subsequently divided using a linear stapler. Next, the ligament of Treitz (suspensory ligament of the duodenum) was divided to ensure there was no tumor encasement of the superior mesenteric artery (SMA), and the distal duodenum and proximal jejunum were divided using an endoscopic linear stapler approximately 10-15 cm from the ligament of Treitz. A Kocher maneuver was then performed by retracting the duodenum medially and the meticulous dissection was carried out until the retroduodenal part of the inferior vena cava (IVC) was exposed sufficiently, and a gauze piece was used as a landmark for safe dissection between the head of the pancreas and the IVC. Considerably, the neck of the pancreas was transected using a harmonic scalpel, with the main pancreatic duct being cut with scissors. Likewise, the head of the pancreas was carefully detached from PV-SMV, and further dissection of the uncinate process was carried on. Veins from the pancreatic head and uncinate process to PV-SMV were cautiously identified, ligated, and divided. Additionally, the first jejunal artery and inferior pancreaticoduodenal artery were also isolated, ligated, and divided. Similarly, dissection of the surrounding adipose and lymphatic tissues was carried out from caudad to cephalad in the mesopancreas region and along the SMA, taking care not to breach the arterial sheath (**Figure 3**). In cases where the tumor involved major veins, vascular resection and reconstruction were performed to ensure a negative margin.

#### 2.3.2 Gastrointestinal reconstruction

**Figure 4:**
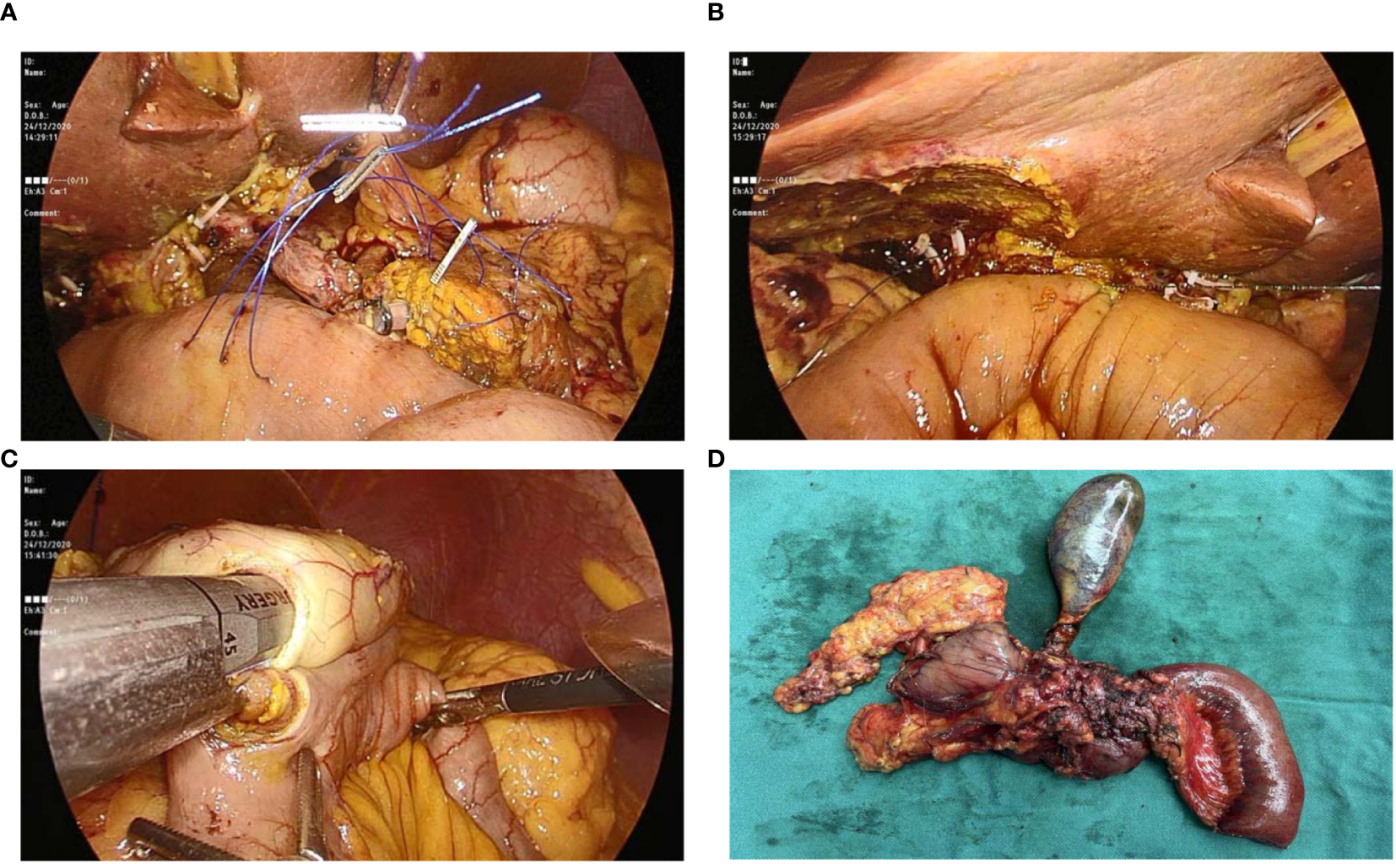
Figure showing: **(A)** Pancreaticojejunostomy with stent placement; **(B)** Hepaticojejunostomy; **(C)** Gastrojejunostomy; and **(D)** Specimen after removal from abdomen.

Our institution adheres to the Child’s technique for gastrointestinal reconstruction following LPD. Briefly, to perform a pancreaticojejunostomy, the procedure was commenced by revealing the pancreatic stump and deftly implanting a silicone catheter as an internal stent into the main pancreatic duct (MPD) selected for optimal fit, secured with a 4-0 prolene suture. A small incision on the jejunal stump, about 5 cm from its sealed end, was complemented by a purse-string suture using the 4-0 prolene suture, where the other end of the silicone stent was then precisely inserted into the lumen of the jejunum, ensuring a snug fit (**Figure 4A**). For cases where the MPD diameter was over 6 mm, the silicone stent was not routinely employed. The anastomosis was meticulously completed using the modified Blumgart technique, ensuring a duct-to-mucosa connection in every instance.

For hepaticojejunostomy, a 4-0 PDS continuous suture was utilized on the posterior wall, with interrupted sutures on the anterior wall (**Figure 4B**). When faced with a common hepatic duct wider than 12 mm, a continuous suture was applied to both walls to reinforce the anastomosis. Finally, the gastroenterostomy was positioned at a calculated distance of 40-45 cm from the hepaticojejunostomy, employing a linear cutter stapler for a side-to-side anastomosis (**Figure 4C**).

Following the precise formation of the anastomoses, we diligently secured hemostasis using vascular clips and prolene sutures to ensure no bleeding occurred. The surgical specimen was then gently removed from the abdominal cavity through a pfannenstiel incision, carefully placed into a sterile plastic bag to preserve aseptic technique (**Figure 4D**). Finally, the abdominal cavity was rinsed with warm water, and three passive-drainage tubes were placed near the operative and anastomosis sites through the port-site incisions, specifically adjacent to the anterior pancreaticojejunostomy, hepaticojejunostomy, and gastrointestinal reconstruction areas.

### 2.4 Postoperative care

Following LPD, all patients were cared for in accordance with our center’s established protocol. The routine administration of octreotide to all patients post-surgery aims to mitigate the risk of potential Postoperative Pancreatic Fistula (POPF). Additionally, our Enhanced Recovery After Surgery (ERAS) protocol emphasizes early mobilization and nutritional intake. Specifically, if the nasogastric drainage output was below 300 ml per day, the tube was removed on the initial postoperative day (POD). The amylase levels in the drainage fluid from each tube were monitored on POD 3 and POD 5, with additional checks as needed. On POD 7, an abdominal CT scan was conducted, and drains were removed when the output volume was consistently less than 50 ml for two consecutive days, provided there were no signs of fever, bleeding, or infection.

### 2.5 Postoperative follow-up

A dedicated team conducted follow-up with all patients within 30 days post-initial surgery, utilizing outpatient clinics, telephone calls, or other communication methods, with all feedback documented in the individual patient’s clinical record. The follow-up primarily focused on the patient’s postoperative recovery progress, the occurrence of any unplanned reoperations, the reasons for such reoperations if they occurred, and the time interval between the initial surgery and any reoperation.

### 2.6 Definitions

The International Study Group of Pancreatic Surgery (ISGPS) criteria were utilized to classify POPF (13), postpancreatectomy hemorrhage (PHH) (14), DGE (15), and chyle leakage (16).

Bile leakage was identified by a threefold increase in the bilirubin concentration in the drain fluid compared to serum bilirubin levels on/or after POD 3, or by the necessity for intervention (radiologic or operative) due to biliary collections or bile peritonitis (17). Furthermore, hyperbilirubinemia was characterized by a serum total bilirubin level reaching 171.0 μmol/L or higher (18). Additionally, an abdominal infection was confirmed when microorganisms were identified in the drainage fluid within the first week following LPD.

Unplanned reoperation was defined as the need for a second surgical procedure to manage complications related to LPD within 30 days of the same hospital admission. Hospital mortality referred to deaths occurring within 30 days of the same admission following LPD. The severity of surgical complications was classified according to Clavien–Dindo classification (19).

Intraoperative bleeding among LPD patients in our study was quantified, with a median blood loss volume of 120 ml. The cutoff values for the bile duct diameter and tumor size were established at the median measurements of 10 cm and 2.6 cm, respectively, for all patients included in the study. A pre-operative serum prealbumin level below the threshold of 0.15 g/L was considered low, suggesting a potential nutritional deficiency.

The assessment of the pancreatic duct’s diameter was a two-step process involving both pre-operative imaging to estimate its size and direct intraoperative measurement for precision. Additionally, the texture of the pancreas was determined by the surgeon’s tactile impression during surgery, identifying it as either soft or hard, and this assessment was later validated by histopathological examination reports. These classifications and measurements were integral to the study’s methodology, ensuring standardized criteria for evaluation.

### 2.7 Statistical analysis

SPSS 25.0 (IBM Corp., Armonk, NY) statistical software was used for data analysis. Continuous variables following normal distribution are presented as mean ± SD (x ± SD) and were analyzed using independent samples t-test. For continuous variables that do not adhere to a normal distribution, are expressed as medians with interquartile ranges and were compared using the Mann–Whitney U test. Similarly, categorical variables are presented as numbers (n) and percentages (%) and were evaluated using Pearson’s χ2 or Fisher’s exact test, as appropriate. Finally, the factors associated with unplanned reoperation within 30 days after LPD were evaluated by univariate and multivariate logistic regression with odds ratios (OR) and 95% Confidence interval (CI). P < 0.05 was considered statistically significant.

## 3 Results

### 3.1 General information

All 311 patients, comprising of 169 males and 142 females with an average age of 66.2 ± 10.7 (20– 92) years and BMI of 22.9 ± 3.04 kg/m2, successfully underwent LPD. Among them, 153 patients had hypertension, 71 had diabetes, and 15 patients suffered from liver cirrhosis. Postoperative pathology for all 311 patients revealed the following: 46 cases of intraductal papillary mucinous neoplasm (IPMN), 86 cases of pancreatic cancer, 48 cases of distal bile duct cancer, 53 cases of duodenal papillary cancer, 28 cases of ampullary cancer, 13 cases of pancreatic serous cystadenoma, 7 cases of gastrointestinal stromal tumors, 5 cases of pancreatic neuroendocrine tumors, 1 case of duodenal neuroendocrine tumor, 1 case of diffuse large B-cell lymphoma, 1 case of chronic pancreatitis, and 22 cases of various other benign tumors.

### 3.2 The rate and causes of unplanned reoperation within 30 days after LPD, and the time interval between two surgeries

Out of 311 patients who underwent LPD, a follow-up conducted within 30 days post-surgery revealed that 23 patients, comprising 7.4% of the total, required an unplanned reoperation within this period (**Table 1**). The leading cause for reoperation was postoperative bleeding, observed in 19 patients (82.6%). Early postoperative bleeding was noted in 4 cases, attributed to detachment of the hemostatic clamp at the gastroduodenal artery stump in two instances, incomplete hemostasis at the pancreatic transection site in one case, and at the stump of a portal vein branch in another. The remaining 15 patients experienced late bleeding, with causes including grade B pancreatic fistulas in seven cases, grade C fistulas in two cases, abdominal infections in two cases, pseudoaneurysm rupture in three cases, and hemostatic clip detachment in one case.

**Table 1:** Unplanned reoperation within 30 days after laparoscopic pancreaticoduodenectomy.

SN	Sex	Age (years)	Pathological diagnosis	Time since first operation (days)	Reason for reoperation	Outcome
1	Male	79	Distal bile duct cancer	8	Hemorrhage from the stump of the dorsal pancreatic artery.	Recovered
2	Male	74	Distal bile duct cancer	4	Massive intraperitoneal hemorrhage	Died
3	Male	62	Duodenal papillary cancer	9	Colonic fistula	Recovered
4	Male	42	Duodenal papillary cancer	14	Hemorrhage from the stump of the right gastric artery, rupture of the gastrointestinal anastomosis with bleeding, and leakage at the bilioenteric anastomosis.	Recovered
5	Male	55	Pancreatic cancer	7	Colonic fistula	Recovered
6	Male	63	IPMN	0	Hemorrhage from the portal vein and its branches.	Recovered
7	Male	72	Ampullary cancer	13	Hemorrhage from the stump of the dorsal pancreatic artery, bleeding from the GDA, and leakage at the bilioenteric anastomosis.	Recovered
8	Female	77	IPMN	2	Hemorrhage in the mesentery of the afferent loop.	Recovered
9	Female	70	Distal bile duct cancer	2	Hemorrhage from the proper hepatic artery.	Recovered
10	Male	58	Ampullary cancer	8, 14	First episode: bleeding from the GDA and leakage at the gastrointestinal anastomosis; second episode: hemorrhage from a pseudoaneurysm of the proper hepatic artery.	Died
11	Female	57	Duodenal papillary cancer	0	Bleeding from the GDA.	Recovered
12	Male	50	Duodenal papillary cancer	1	Hemorrhage at the pancreatic transection margin.	Recovered
13	Male	67	IPMN	23	Obstruction of the afferent loop.	Recovered
14	Female	74	Distal bile duct cancer	30	Intra-abdominal infection	Recovered
15	Male	60	Duodenal papillary cancer	20, 25	Two postoperative episodes of bleeding due to rupture of the GDA stump.	Recovered
16	Male	73	Duodenal papillary cancer	27, 29	On the 27th postoperative day: hemorrhage at the pancreatojejunal anastomosis and hepatic artery rupture; on the 29th postoperative day: hemorrhage from the common hepatic artery.	Recovered
17	Male	71	Distal bile duct cancer	9	Biliary leakage and biliary-enteric anastomosis bleeding.	Recovered
18	Female	66	Duodenal papillary cancer	6	Massive intraperitoneal hemorrhage.	Recovered
19	Female	65	Duodenal papillary cancer	27	Massive intraperitoneal hemorrhage.	Recovered
20	Female	61	Distal bile duct cancer	1	Massive intraperitoneal hemorrhage.	Recovered
21	Female	61	Duodenal papillary cancer	10	Hemorrhage from a pseudoaneurysm of the left hepatic artery.	Recovered
22	Female	68	Distal bile duct cancer	2	Hemorrhage from a pseudoaneurysm of the left hepatic artery.	Recovered
23	Male	69	Distal bile duct cancer	11	Bleeding from the portal vein.	Recovered

PMN, Intraductal Papillary Mucinous Neoplasm; GDA, Gastroduodenal Artery.

In addition to bleeding, other causes for reoperation included two cases of combined biliary-enteric anastomotic leakage, one case of gastrointestinal anastomotic leakage, two cases of colonic leakage, one further case of abdominal infection, and one case of afferent loop obstruction after LPD. The timing of reoperations varied, with procedures carried out within 0 to 7 days for 10 patients, between 8 to 14 days for 8 patients, and between 15 to 30 days for 5 patients. Notably, 3 of these patients required reoperation twice.

### 3.3 Postoperative outcomes of the patients within 30 days after surgery in the reoperation group and non-reoperation group

Among the 311 patients who underwent LPD, they were categorized into two groups based on receiving an unplanned surgery within 30 days post-operation: the reoperation group with 23 cases and the non-reoperation group with 288 cases. The rate of complications and mortality was significantly higher in the reoperation group, at 73.9% (17 out of 23) compared to 37.5% (108 out of 288), and 8.7% (2 out of 23) compared to 0.3% (1 out of 288), respectively. Additionally, the reoperation group experienced a longer hospital stay, averaging 31.0 ± 18.4 days, in contrast to the non-reoperation group, which averaged 17.5 ± 8.1 days.

### 3.4 Risk factors for unplanned reoperation within 30 days after LPD

While comparing the reoperation and non-reoperation groups, the univariate analysis identified several factors significantly associated with a higher risk of unplanned reoperation within 30 days after LPD (**Table 2**). These factors included diabetes (P = 0.014), liver cirrhosis (P = 0.001), elevated C-reactive protein (CRP) levels on POD-3 (P = 0.002) and POD-7 (P < 0.001), a pre-operative serum prealbumin level below 0.15 g/L (P = 0.001), prolonged operation time (P = 0.025), intraoperative bleeding exceeding 120 ml (P = 0.009), vascular reconstruction (P = 0.001), a soft pancreas texture (P < 0.001), and a main pancreatic duct diameter of ≤3 mm (P = 0.011).

**Table 2:** Univariate analysis of risk factors for unplanned reoperation within 30 days after laparoscopic pancreaticoduodenectomy.

Variables	Reoperation group(n= 23)	Non-reoperation group(n= 288)	X^2^	P-value
Age (years)a	64.9 ± 8.9	66.3 ± 10.8	0.59	0.556
Maleb	14 (60.9)	155 (53.8)	0.43	0.514
Body Mass Index (kg/m2)a	23.7 ± 3.3	22.8 ± 3.0	-1.39	0.165
Diabetesb	10 (43.5)	61 (21.2)	6.01	0.014
Hypertensionb	7 (30.4)	146 (50.7)	3.50	0.061
Cirrhosisb	5 (21.7)	10 (3.5)	11.76	0.001
History of upper abdominal surgeryb	5 (21.7)	36 (12.5)	0.88	0.347
Reduction in preoperative jaundiceb	2 (8.7)	28 (9.7)	<0.001	1.000
Preoperative total bilirubinb			0.25	0.620
≥ 171 µmol/L	6 (26.1)	56 (19.4)		
< 171 µmol/L	17 (73.9)	232 (80.6)		
Neoadjuvant chemotherapyb	0 (0.0)	2 (0.7)	<0.001	1.000
Preoperative serum prealbumin levelb			10.35	0.001
≥ 0.15 g/L	11 (47.8)	224 (77.8)		
< 0.15 g/L	12 (52.2)	64 (22.2)		
CRP (mg/L)				
POD-1	40.1 (23.2,75.0)	30.1 (17.2,52.6)	-1.68	0.093
POD-3	136.0 (110.0,161.5)	91.1 (55.3,131.2)	-3.05	0.002
POD-7	92.3 (77.5,92.3)	49.9 (18.5,79.7)	-4.04	<0.001
ASA gradeb			0.01	0.915
> 2 grade	1 (4.3)	21 (7.3)		
≤ 2 grade	22 (95.7)	267 (92.7)		
Operation time (hrs)a	7.4 ± 2.2	6.3 ± 1.4	-2.39	0.025
Vascular reconstructionb	7 (30.4)	21 (7.3)	11.24	0.001
Use of linear staplerb	21 (91.3)	281 (97.6)	1.16	0.281
Intraoperative blood lossb			6.92	0.009
> 120 ml	16 (69.6)	119 (41.3)		
≤ 120 ml	7 (30.4)	169 (58.7)		
Pancreatic textureb			16.83	<0.001
Soft	17 (73.9)	91 (31.6)		
Hard	6 (26.1)	197 (68.4)		
Diameter of main pancreatic ductb			6.52	0.011
> 3 mm	11 (47.8)	210 (72.9)		
≤ 3 mm	12 (52.2)	78 (27.1)		
Diameter of common bile ductb			1.72	0.190
≥ 10 mm	17 (73.9)	173 (60.1)		
< 10 mm	6 (26.1)	115 (39.9)		
Tumor typeb			0.27	0.606
Benign	6 (26.1)	90 (31.2)		
Malignant	17 (73.9)	198 (68.8)		
Tumor sizeb			1.35	0.245
> 2.6 cm	14 (60.9)	139 (48.3)		
≤ 2.6 cm	9 (39.1)	149 (51.7)		
Number of lymph nodes dissecteda	11.3 ± 8.6	11.6 ± 7.4	-0.45	0.656

ASA, American Society of Anesthesiologists; CRP, C-reactive protein.

a expressed as Mean ± SD;

b expressed as percentage (%).

Furthermore, multivariate analysis confirmed that a pre-operative serum prealbumin level below 0.15 g/L (OR = 3.519, 95% CI: 1.167-10.613, P = 0.025), elevated CRP levels on POD-7 (OR = 1.013, 95% CI: 1.001-1.026, P = 0.029), vascular reconstruction (OR = 9.897, 95% CI: 2.405-40.733, P = 0.001), a soft pancreatic texture (OR = 5.243, 95% CI: 1.628-16.885, P = 0.005), and a main pancreatic duct diameter of ≤3 mm (OR = 3.462, 95% CI: 1.049-11.423, P = 0.041) are independent risk factors for unplanned reoperation in LPD patients within 30 days after surgery (**Table 3**).

**Table 3:** Multivariate logistic regression analysis for unplanned reoperation within 30 days after laparoscopic pancreaticoduodenectomy.

Variables	β	S.E	Wald	P value	OR	95% CI
Diabetes	0.956	0.573	2.791	0.095	2.602	0.847~7.993
Cirrhosis	0.855	0.838	1.042	0.307	2.352	0.455~12.153
Preoperative serum prealbumin level < 0.15 g/L	1.258	0.563	4.992	0.025	3.519	1.167~10.613
CRP (POD-3)	0.000	0.006	0.002	0.961	1.000	0.989~1.011
CRP (POD-7)	0.013	0.006	4.781	0.029	1.013	1.001~1.026
Operation time	0.142	0.186	0.581	0.446	1.152	0.800~1.658
Intraoperative blood loss > 120 ml	0.274	0.591	0.216	0.642	1.316	0.413~4.191
Vascular reconstruction	2.292	0.722	10.083	0.001	9.897	2.405~40.733
Soft pancreas	1.657	0.597	7.712	0.005	5.243	1.628~16.885
Diameter of the main pancreatic duct ≤ 3 mm	1.242	0.609	4.157	0.041	3.462	1.049~11.423

β, Regression coefficient; S.E., Standard error of regression coefficient; Wald, Wald chi-square value; CI, Confidence interval; OR, Odds ratio.

## 4 Discussion

As laparoscopic surgery advances and the emphasis on early intervention in the peri-operative period grows, the 30-day mortality rate following LPD has consistently remained low, typically ranging from 0% to 4.8% (7, 8). This is corroborated by our study, which reports a rate of 0.9% (3 out of 311 cases). Despite these advancements, the technical intricacies of LPD, which include complex dissection and reconstruction, continue to pose a high risk of postoperative complications. In some instances, these complications are so severe that they necessitate reoperation after conservative measures fail. Unplanned reoperations have profound implications for both patients and the healthcare system. They impose considerable physical and psychological stress on patients and can also exacerbate the workload of medical professionals, potentially diminishing the quality of care provided. It is therefore crucial to reduce the likelihood of unplanned reoperations through meticulous pre-operative planning, proactive management of risk factors, precise surgical techniques, and attentive postoperative monitoring. However, the literature on the causes and risk factors for unplanned reoperations following LPD is limited.

Historical data suggests a reoperation rate of 6.8% to 15.4% after LPD (7, 20) with serious complications such as postoperative hemorrhage, pancreatic fistulas, bile leaks, and intra-abdominal infections being the primary drivers of these unplanned reoperations (20). In line with these findings, our study observed an unplanned reoperation rate of 7.25% (23/311) within 30 days of LPD, with the majority of these cases 82.6% (19/23) attributed to postoperative bleeding (including 3 cases biliary-enteric anastomotic leakage and 1 case of gastrointestinal anastomotic leakage), along with 2 cases of colonic leakage, 1 case of abdominal infection, and 1 case of afferent loop obstruction.

Postoperative hemorrhage stands as a grave complication following pancreaticoduodenectomy, with studies indicating an occurrence rate of 4% to 18% in open procedures and a notably higher 13% to 24% in laparoscopic pancreaticoduodenectomy (LPD), where the associated mortality rate can soar to 30% (21–24). Our study mirrors these findings, with 7.1% (22/311) of LPD patients experiencing postoperative bleeding. The primary sites of bleeding were identified as the gastroduodenal artery stump, right gastric artery stump, dorsal pancreatic artery stump, remnant of the pancreatic stump, portal vein and branches of the proper hepatic artery, the digestive tract reconstruction anastomosis site, and hepatic artery pseudoaneurysm rupture. The ISGPS has established a classification system for postoperative bleeding, categorizing it into three distinct grades: A, B, and C. Grade A encompasses mild bleeding that occurs early, typically within the first 24 hours. Grade B includes significant early bleeding or mild bleeding that is delayed, usually after more than 24 hours. Grade C refers to major bleeding that is delayed. Early bleeding is often associated with the patient’s peri-operative coagulopathy, inadequate intraoperative hemostasis, suboptimal surgical techniques in vascular ligation, and incorrect application of linear cutting closure devices. Delayed bleeding, on the other hand, is predominantly linked to the development of abdominal pseudoaneurysms, pancreatic fistulas, bile leaks, and abdominal infections (14). In this study, early postoperative bleeding was observed in 4 out of 19 patients, while the remaining 15 experienced late bleeding. The causes of early bleeding included detachment of the hemostatic clamp in two instances at the gastroduodenal artery stump, incomplete hemostasis at the pancreatic transection site in one case, and at the stump of portal vein branch in another. Similarly, the reasons for late bleeding were attributed to grade B pancreatic fistulas in seven cases, grade C fistulas in two cases, abdominal infections in two cases, pseudoaneurysm rupture in three cases, and hemostatic clip detachment in one case. Notably, three patients with late bleeding had postoperative hemorrhage even after an unplanned reoperation. Our findings highlight that pancreatic fistulas are the primary cause of delayed postoperative bleeding, posing a significant challenge to manage once they occur. The etiology of postoperative bleeding from pancreatic fistulas may stem from the corrosive nature of pancreatic secretions on blood vessels, and makes them difficult to heal (25). As stated earlier, postoperative bleeding was the leading cause of unplanned reoperation following LPD in this study. The condition can deteriorate rapidly, and without timely detection and intervention, severe complications such as shock can arise, leading to detrimental outcomes for the patient. Therefore, to mitigate the need for reoperation due to postoperative bleeding, early intervention and management are crucial. It is recommended that prior to LPD, patients should undergo thorough pre-operative evaluations to identify and promptly correct conditions like hypertension and coagulation disorders. Additionally, enhancing nutritional support can improve patients’ overall health. Moreover, precise anatomical knowledge, careful dissection, and the correct application of surgical tools, including ultrasonic scalpels and linear cutters, can ensure complete hemostasis and reduce the risk of pseudoaneurysm formation. Postoperatively, surgeons should closely monitor the characteristics of the drainage fluid and regularly check amylase levels. Besides, prophylactic use of somatostatin analogs like octreotide, along with appropriate antibiotics, is advised for all patients to prevent potential pancreatic fistulas and abdominal infections (26). Finally, for any identified arterial bleeding, prompt use of digital subtraction angiography is essential for diagnosing and managing the bleeding source effectively.

On univariate analysis, this study found that factors like diabetes, liver cirrhosis, raised CRP on POD-3 and POD-7, pre-operative serum prealbumin level <0.15 g/L, prolonged operation time, intraoperative bleeding >120 ml, vascular reconstruction, a soft pancreas, and a main pancreatic duct diameter ≤3 mm were significantly associated with a higher risk of unplanned reoperation within 30 days following LPD. However, upon multivariate analysis, only a pre-operative serum prealbumin level <0.15 g/L, elevated CRP on POD-7, vascular reconstruction, a soft pancreas, and a main pancreatic duct diameter ≤3 mm were independent risk factors for unplanned reoperation.

Serum prealbumin a liver-synthesized globular protein (27), is often low in patients with severe malnutrition, which can delay wound healing and is associated with acute inflammation, definite infection, and production of large numbers of cytokines (28, 29). In particular, earlier studies have reported that low serum prealbumin is associated with poor prognosis in patients with burn injuries, acute respiratory distress syndrome, and cardiac diseases (30–32). Similarly, CRP is an inflammatory marker that increases during an acute inflammatory response and has been correlated with the development of clinically relevant POPF (33), anastomotic leakage (34), intra-abdominal infection, and surgical site infections (35).

Likewise, vascular reconstruction during LPD is a crucial aspect of the procedure that can be associated with certain risks, and might potentially lead to the need for reoperation after LPD. Particularly, vascular reconstruction during LPD is technically challenging and requires high level of surgical skills and the risks of morbidity and mortality increase notably with the extent of vascular resection. Any difficulties during this phase such as improper anastomosis, or unrecognized bleeding could necessitate reoperation. Moreover, vascular injury during LPD can lead to complications such as venous thrombosis, formation of pseudoaneurysm and bleeding. As the primary goal of vascular resection during LPD is to achieve R0 resection for pancreatic tumors. However, complications related to vascular reconstruction may impact the oncological outcomes and might also require additional surgery (36). Besides, other factors like patient comorbidities, the complex nature of the tumor, and use of vascular grafts for vascular reconstruction can also influence the outcome of vascular reconstruction, and thereby increase the risk of reoperation (3, 36). Ultimately, while vascular reconstruction is an essential step in LPD, it carries inherent risks that might lead to reoperation. Thus, we suggest, meticulous surgical technique, careful postoperative care, and vigilant surveillance with postoperative imaging such as CT and MRI for early detection of any bleeding, vascular thrombosis or stenosis are important in minimizing the risk of reoperation and ensuring the best possible outcomes for patients.

Lastly, the presence of a soft pancreas and a pancreatic duct with a diameter ≤3 mm are another independent risk factor that could necessitate an unplanned reoperation. This is likely due to the increased possibility of developing pancreatic fistula. A soft pancreas is more prone to leakage after pancreaticojejunostomy, as the soft texture of pancreas makes it challenging to achieve secure anastomosis. Similarly, a main pancreatic duct diameter ≤3 mm makes anastomosis technically difficult, and a risk of leakage increases, which may need reoperation to manage POPF. These findings align with the research conducted by Schuh et al. (37) who mentioned that a soft pancreas and a main pancreatic duct diameter ≤3 mm were significantly associated with the occurrence of pancreatic fistula after pancreatic surgery.

Our study, while valuable, presents several limitations that must be acknowledged. Its retrospective nature inherently subjects it to potential biases due to reliance on historical data. The data, spanning from 2017 to 2024, may reflect variations in surgical techniques, which could impact the uniformity of our analysis. Although all procedures were conducted by surgeons proficient in LPD, the inconsistency among surgical teams and the possible influence of a learning curve introduce additional variability. Furthermore, our electronic medical record system’s limitations may have resulted in incomplete data capture, and our study was restricted to a 30-day postoperative follow-up, potentially narrowing the scope of our findings. Nonetheless, the study’s significance is bolstered by its extensive dataset from a single center, offering a more focused perspective. We have also conducted a thorough analysis of a wide range of clinically relevant variables that could influence the risk of unplanned reoperation following LPD, thereby enhancing the study’s clinical relevance and applicability.

In conclusion, the study highlights the significant impact of postoperative bleeding on the rate of unplanned reoperations following LPD. Independent risk factors contributing to unplanned reoperations following LPD include a pre-operative serum prealbumin level below 0.15g/L, elevated C-reactive protein (CRP) levels on the POD-7, soft pancreatic texture, and a main pancreatic duct diameter of ≤3 mm. The findings emphasize the importance of comprehensive peri-operative management, with a focus on pre-operative planning and postoperative monitoring, especially for patients with these identified risk factors. By proactively addressing these factors, the study suggests that it is possible to significantly reduce the likelihood of unplanned reoperations and enhance patient outcomes post-LPD.

## 5 Institutional protocol considerations

The findings of our study underscore the importance of stringent pre-operative assessment, intra-operative precision, and vigilant post-operative care to minimize the risk of unplanned reoperations following LPD. As a direct response to our observations, several modifications to our institutional protocols are currently being contemplated.

Firstly, we recognize the critical role of pre-operative nutritional assessment. We are looking to implement a more thorough evaluation process to identify and correct any nutritional deficiencies, such as low serum prealbumin levels, which can affect wound healing and increase the risk of complications.

In light of the correlation between vascular reconstruction and the need for reoperation, we are focusing on bolstering the training and education of our surgical staff. This includes the use of simulation-based learning to perfect vascular anastomosis techniques. Additionally, we aim to standardize post-operative care pathways, with a particular emphasis on the monitoring of drainage fluid to ensure early detection and intervention for bleeding or anastomotic leaks.

Considering the impact of pancreatic texture and duct diameter on reoperation risk, we are refining our patient selection criteria for LPD. This may involve considering alternative surgical approaches for patients with a soft pancreas or smaller pancreatic duct diameters, to minimize the risk of complications.

Lastly, to address the observed rates of postoperative bleeding, we are exploring the integration of advanced imaging and interventional radiology services into our post-operative care protocols. This will allow for rapid and effective management of bleeding complications, enhancing patient safety and outcomes.

These contemplated changes reflect our commitment to align our practices with the latest evidence-based findings, with the aim of improving patient safety, optimizing healthcare resource utilization, and enhancing the overall quality of care for LPD patients.

## Data Availability

The original contributions presented in the study are included in the article/supplementary material. Further inquiries can be directed to the corresponding authors.
